# Supplementary material for: Reduction of Tumor Growth with RNA-Targeting Treatment of the NAB2–STAT6 Fusion Transcript in Solitary Fibrous Tumor Models
Source: Cancers (Basel). 2023 Jun 9;15(12):3127. doi: 10.3390/cancers15123127 (PMC10296122; doi:10.3390/cancers15123127)
Supplement: Supplementary file 1 [file cancers-15-03127-s001.zip › Supplementary_Materials.pdf]

Supplementary materials

General cloning protocols

The Q5 High-Fidelity 2X Master Mix (New England Biolabs, catalog number: M0492) was used for all polymerase chain reactions (PCR) according to the manufacturer's protocol. All oligonucleotides were ordered from Sigma-Aldrich and were listed in **Supplementary Table S1**. The plasmids were constructed using PCR amplification, restriction digest (all restriction enzymes were ordered from New England Biolabs), and ligation with T4 DNA ligase (New England Biolabs, catalog number: M0202). Gel purification and PCR purification were performed with QIAquick Gel Extraction kit (Qiagen, catalog number: 28707) and PCR Purification kit (Qiagen, catalog number: 28104). Transformations were performed using NEB 5-alpha electrocompetent *Escherichia Coli* cells (New England Biolabs, catalog number: C2987). The minipreps were performed using QIAprep Spin Miniprep kit (Qiagen, catalog number: 27106). The final plasmids were confirmed by both restriction enzyme digestions and direct Sanger sequencings.

DNA constructs

PCMV-spCas9-U6-sgRNA.left: The U6-sgRNA.left sequence was first PCR amplified from PCMV-spCas9-U6-sgRNA.PUF (unpublished results) using primers P7 and P8, and the PCR product was subsequently subjected to the second round PCR using primers P7 and P9. The PCR product was then cloned into the PCMV-spCas9-U6-sgRNA.PUF vector using KpnI and XbaI sites.

PCMV-spCas9-U6-sgRNA.right: The U6-sgRNA.right sequence was first PCR amplified from PCMV-spCas9-U6-sgRNA.PUF (unpublished results) using primers P7 and P10, and the PCR product was subsequently subjected to the second round PCR using primers P7 and P9. The PCR product was then cloned into the PCMV-spCas9-U6-sgRNA.PUF vector using KpnI and XbaI sites.

Homologous Recombination (HR) repair template: The NAB2-STAT6 left homologous arm was PCR amplified using HCT116 genomic DNA as the template and primers P11 and P12. The PCR product was then cloned into the PGK1-Hygromycin resistance gene vector (unpublished results) using ClaI and FseI sites. The resulting plasmid was named Step1. Next, the NAB2-STAT6 right homologous arm was PCR amplified using HCT116 genomic DNA as the template and primers P13 and P14. The PCR product was then cloned into the Step1 plasmid using NotI and MScI sites. The resulting plasmid was named Step2. Next, to add new restriction enzyme sites for later knock-in sequences, a PCR reaction was performed using Step2 plasmid as the template and primers P15 and P16. The PCR product was then cloned into Step2 plasmid using ClaI and FseI sites. The resulting plasmid was named Step3. Next, one portion of the knock-in sequence was PCR amplified using HCT116 genomic DNA as the template and primers P17 and P18. The PCR product was then cloned into Step3 plasmid using XhoI and SanDI sites. The resulting plasmid was named Step4. Next, another portion of the knock-in sequence was PCR amplified using HCT116 genomic DNA as the template and primers P19 and P20. The PCR product was then cloned into Step4 plasmid using FseI and SanDI sites. The resulting plasmid was named Step5. Finally, the last portion of the knock-in sequence was synthesized at Genewiz and cloned into Step5 plasmid using MluI and XhoI sites.

PCMV-CasRx-U6-sgRNA.NAB2STAT6: The GFAP-CasRx-U6-sgRNA was ordered from Addgene (catalog number: 154001). The CMV promoter sequence was PCR amplified from D1 (unpublished results) using primers P27 and P28. The PCR product was then cloned into GFAP-CasRx-U6-sgRNA using XbaI and SgrAI sites. The resulting plasmid was named NS4.1.1. Next, the U6-sgRNA.NAB2STAT6 sequence was first PCR amplified from NS4.1.1 using primers P29 and P30, and the PCR product was subsequently subjected to the second round PCR using primers P2 and P31. The PCR product was then cloned into NS4.1.1 vector using BglII and EcoRI sites.

PCMV-ATG-target.NAB2STAT6-YFP-PEST: The target.NAB2STAT6-YFP-PEST was PCR amplified from B12 (unpublished results) using primers P32 and P33. The PCR product was then cloned into B12 using NheI and XmnI sites.

PCMV-CasRx-U6-sgRNA.AAVS1: The U6-sgRNA.AAVS1 sequence was PCR amplified from NS4.1.1 using primers P29 and P34, and the PCR product was subsequently subjected to the second round PCR using primers P2 and P35. The PCR product was then cloned into NS4.1.1 vector using BglII and EcoRI sites.

Homologous Recombination (HR) repair template sequence:
TCGACATCGATGCCAGCCCATGGGCTATGGAGCCGACACATCCTGCAGCAGACACTGATGGACGAGGGGCTGCG
GCTCGCCCGCCTCGTCTCCCACGACCGCGTGGGCCGCCTCAGCCCCTGTGTGCCTGCGAAGCCACCTCTCGCAGGT
GAGGCAGCCAGCAGTGCTGTCCCCAAGCACCCCGGCCACTCAGGCCCCACACAGCAATTCTGGTGTCCTGGGGCC
AGGTGGGAGGAAGAGGGATATAGTCGTCAGAGAGGGCAGTAGGATGGCTGGGCTCCAGCCAGCCAGGCCTTGG
GGTGCAGAGAGGCAGACCCTGCCGAGCTGCTCCCACCATGGAAAGTCTGGTCCTAGTGTAGATCTGTGAAAGGG
GAACTGGGGAATGGGGGACAGAGAAAAGAGGATGAAGGAGCAGGAAGGAGGAGCATCAATAAGGGAAGCTC
AGATGGCACTTCCACCAACCCAGGAGTGGGGAGCAGGCTAGGAGTTGTGGGTATGGGGATGGGGATGCCCAGCT
TGGAAGCAGAGGAGCCCAAAGCAGTGATGCTTTGTGTGTGGAAGGGGGAAGAGGGAGTGGGCAGGCGGAGGG
CCGGGCAGCCGCTGGGCTGAGAGTGACGCAGCAGACAAAGCCACCAACCCTGGCTTGGTATTTTTAAACTGTGCG
TGGGTGGGAAGTGGGGGCGGGGACTGGAAGGGGGGCTCTTCTTGAGGGAGGAGCTCGGAGGCTGGCTTCCTGT
TCTCCCTCTGATTTGGCTACCAAAAAACTTGGGGGGCCATGGTAGAGTGGCCTGGGAGGGGCCTGGGAGGGGGC
CAGGGATGAGTGGGGGTGGCAGAGCTTGGGAAGGGGGGAGGGGAGAGGTCAGAGCCAAACCACCCCCGCAGC
TCCTGACAGAACCAGAAATTCCAGCGCACGCGTATTGGGATCAGTACACGCGTTCTGATTGGTTCCTGTCACCCAC
TTCCTCCCTGCTCCCCGCTGCCCTCCTCTCCCTGCCCGCGTGTCATCCTGATGTCACTCCCTATTTCATAGCTGTGCTT
CTCTTACTTCCCCATGATCCATGCCCACCTTTTCCACCTCCCTTCCTCCCTAACCCCAGAGCACTCCATGGCTGTCTTT
TCCTTCTCACAACAGCTGAACAGATGGGTAAGGATGGCAGGGGTTATGTCCCAGCTACCATCAAGATGACCGTGG
AAAGGTGAGTGTGGTGGTATGGACAGTGGGTAGGTCAGGGGCTTAGTGCTTATCTGCAGGAAGGAGGGGTGGC
ATCAACCCTTGGTCAGTCACATGTACCTCCTTCCCTCCTCCAGGGACCAACCACTTCCTACCCCAGAGCTCCAGATG
CCTACCATGGTGCCTTCTTATGACCTTGGAATGGCCCCTGATTCCTCCATGAGCATGCAGCTTGGCCCAGATATGGT
GTAAGGAGCTGGAAAGACAGGAATGGGAGTGGTCTGTGCAGATGGGCTAATCTTAGCATGGGCAGCTGGGAGA
GCTGGCACTGGGGGCTGAACAGGGAATCTTCCTTTCCATGAGAGGGACACCTGTTCAAAAGCAGGGTGTGGTGG
TGTCCAGGAGAAGGGCTGGCATCAGGGGGTCTGTTTTCTTTCCCCAGGCCCCAGGTGTACCCACCACACTCTCACT
CCATCCCCCCGTATCAAGGCCTCTCCCCAGAAGAATCAGTCAACGTGTTGTCAGCCTTCCAGGAGTAAGTGAAAAA
CCTCATGGGGATACCATCCCACTCTAAGGGGGTGGGCATTTGAATTGTTAGAAGAGGCTCTTCTGTGAGAAAGGA
GCAGCAAATGCTAACAGCCTGTCTTCTTCTCTTCTGTCCACTCTAATGAGGGGGTAGTAGTTAAGATCTGGACTGCC
TAGGTTTGAATTCTAGCTCCACCACTTACTGGTTTGGGGCAAATTACTTAGCCTTTGGTGCCTTATCTGCACAATGG
GGGATAATAATGCTAATAATAATAACCTACCTCACTGCATTATTGTGGAGATTAAATGAGTTCATAACACTTAAAAA
GCTGAGCATAGTGCATGGCTCATAGCAAAAGCTGTGTAAGTCCAGTCGTGGATCACTTAATGAAGGAGCATTTTCT
GTCTTTGGCAGTTTCATAATTATGCGAATACCATTGAGTATAATTACACAAACCTAGATGGTATAGACTACTATACA
CTGAGGCTATATTGTGTAGCCTATTGATCCTAGCTTTAAACCCGAGCAGCATGATACTGTTCTGAATAGTATAAGG
AAATAGTAACATAATGGTAAATATTTGTGTGATAGGAATTTTCAGCTTGATTATAATTTTTTTTTTTTGAGACAGGG
TCTCACTCACTGGAGTGCAGTGGTGCGATCTTAGCTCCCTGCAACCTCCGCCTCTTGGGCTCGAGCAATCCTCCTGC
TGTAGTGCACCACGACACTCGGCTAATTCTTTTTTAAGATTTTTCTGCAGACAAGGTCTCACTTACTGCCCAAGCTG
GTCTCAAACTCCTGGGCTTAAGTGATCCTCCCACCTCGGCCTCCCAAAGCGTTAGGATTACAGGCGTGAGTCACTC
TGCCTGGCCTTGATTATAATCTTATGGGACCACTGTGGTCTGTAGTTGACAGAAATGTCGTTAATGTGGTGCATGA
CTGTTATTATTATTTTCTGTCCTGCCCCTGAGAGCCACTGTCACTTCTCTGCTGTATTGGTTTTTGTTTACTCATCTGT
TTTGGCCTTGAAATGGCCTAGACATTTTTCTTCCCGAAGTATGACACTCGGGTGCTTATTAACTTAGTCAAGACACA
ACATCTCCCTTCCCAGAAGGTGAGGCGGGAGTGAGGACTTGGGGACTTAAGAACTACCAAAGTTCAGAGTCCAAA
GAAACATTAGAAATTGGCTAATCCACCCCCATAACACGCACATTTTACAGATGAGAAGACTGAGCTCAGAGCATAG
AAATAGCTTGCCCAGGCCATGACTAAGTCAGGATAAGGAGCTGGAGCTTGTTTCCTCACTCAGTGGTCCTGACTTT
GCACCACTCTGCATTTGCCTAGCCTGCCTTCCTCTAACTGTGCTCTCCCTACTTCCAGGCCTCACCTGCAGATGCCCC
CCAGCCTGGGCCAGATGAGCCTGCCCTTTGACCAGCCTCACCCCCAGTGAGTGACAAAGCCCCTCCTGACCCCATG
TGCCTCTTCTTTCCTGGCCTTGCCCCGCTCTCCTTATTTCCATTGCTGGTTCCTGGCAGGGGCCTGCTGCCGTGCCAG
CCTCAGGAGCATGCTGTGTCCAGCCCTGACCCCCTGCTCTGCTCAGATGTGACCATGGTGGAAGACAGCTGCCTGA
GCCAGCCAGTGACAGCGTTTCCTCAGGGCACTTGGTGAGTGGCAGCTTGGGAGTGGAGGCTGGGTGGCATCTAG
GGGAGTGGGCGCCATGCCTACTCCACTGCTTCTCCCATCTCCTTGCAGGATTGGTGAAGACATATTCCCTCCTCTGC
TGCCTCCCACTGAACAGGACCTCACTAAGCTTCTCCTGGAGGGGCAAGGGGAGTCGGGGGGAGGGTCCTTGGGG

GCACAGCCCCTCCTGCAGCCCTCCCACTATGGGCAATCTGGGATCTCAATGTCCCACATGGACCTAAGGGCCAACC
CCAGTTGGTGATCCCAGCTGGAGGGAGAACCCAAAGAGACAGCTCTTCTACTACCCCCACAGACCTGCTCTGGAC
ACTTGCTCATGCCCTGCCAAGCAGCAGATGGGGAGGGTGCCCTCCTATCCCCACCTACTCCTGGGTCAGGAGGAA
AAGACTAACAGGAGAATGCACAGTGGGTGGAGCCAATCCACTCCTTCCTTTCTATCATTCCCCTGCCCACCTCCTTC
CAGCACTGACTGGAAGGGAAGTTCAGGCTCTGAGACACACCCCAACATGCCTGCACCTGCAGCGCGCACACGCAC
GCACACACACATACAGAGCTCTCTGAGGGTGATGGGGCTGAGCAGGAGGGGGGCTGGGTAAGAGCACAGGTTA
GGGCATGGAAGGCTTCTCCGCCCATTCTGACCCAGGGCCTAGGACGGATAGGCAGGAACATACAGACACATTTAC
ACTAGAGGCCAGGGATAGAGGATATTGGGTCTCAGCCCTAGGGGAATGGGAAGCAGCTCAAGGGACCCTGGGT
GGGAGCATAGGAGGGGTCTGGACATGTGGTTACTAGTACAGGTTTTGCCCTGATTAAAAAATCTCCCAAAGCCCC
AAATTCCTGTTAGCCAGGTGGAGGCTTCTGATACGTGTATGAGACTATGCAAAAGTACAAGGGCTGAGATTCTTC
GTGTATAGCTGTGTGAACGTGTATGTACCTAGGATATGTTAAATGTATAGCTGGCACCTTAGTTGCATGACCACAT
AGAACATGTGTCTATCTGCTTTTGCCTACGTGACAACACAAATTTGGGAGGGTGAGACACTGCACAGAAGACAGC
AGCAAGTGTGCTGGCCTCTCTGACATATGCTAACCCCCAAATACTCTGAATTTGGAGTCTGACTGTGCCCAAGTGG
GTCCAAGTGGCTGTGACATCTACGTATGGCTCCACACCTCCAATGCTGCCTGGGAGCCAGGGTGAGAGTCTGGGT
CCAGGCCTGGCCATGTGGCCCTCCAGTGTATGAGAGGGCCCTGCCTGCTGCATCTTTTCTGTTGCCCCATCCACCG
CCAGCTTCCCTTCACTCCCCTATCCCATTCTCCCTCTCAAGGCAGGGGTCATAGATCCTAAGCCATAAAATAAATTTT
ATTCCAAAATAACAAAATAAATAATCTACTGTACACAATCTGAAAAGAAAGACGCTCTAACTGCTCAGATAGGTGC
TGCGGTCCAGCCCCCAGCTGGAGGAGACCCTGAGTCCAACCCAGGCCTCCCGAGGGGGCCAGTGAAGGGATCCC
ACACCCACCGCCCCTATGTAGGGCAGGGAAGAAATTGCAAAGGACTTGGGGGATAGATGGGAATGGGAGGGCA
AACTGCAGCACTTGTTAAATTAATTAAAGAAACAAACCAGAAGCACAAAAACGGGGAAGGAGAGGGGAGAAGG
AGCAGGTCCAGTGTTCCCAGGCCCCCAATTCTGGGGGCAAATGTTGCCACTTTTAGCTGGACCTTCCCAGGGAAGT
CCCCCTTTCCCCCTTGTCCAAACTGAGTCCAACTGCTCACACCACTGGTGCAAACCTAAAGAGAATGGGAGTGTGT
TGTGTGAGGGAGGGGAAAACCCCAGATGCCCTGGAGGCAGAGTCCTTGCCCCCTCGCTCTGGGCAGGGGTGAGG
GCACCACGCACCCTCCTTGTGTCCTCTCTCCAGGCCTCTGCTTGTTATTGCTTGAAGCCCCACAGTCTTATGCCCATG
GAGCAGGTGGGGAGGCAGGAGAAGGGCAGAGGAAGGATCCAGGGTTGTGACTAGATTCCGGGGATGGAGAGC
GTGGGGGTCTGTGTGAGCCAGAAGTCTGAGGTCCTGGGTCTGAAGACACCAGTCCAACCCTCACTGCCGGCTGGC
CTCAGCCTCCACTTTCACGCTGCTCCTGCGACCCTCCACCAGCGCGGGATGGGGTCCTGGGGCAGGACATCTGTCC
AGCAGCCCTTCCTCGAACTCTGCAGGGAAAAGGGGGCAGTTAGGAGACTGCAGGGTGACTGGCTGCAGACAGAG
GTGGTTGGGAACAGAAGCAGGGGCAAAGAAAGCTGTGAAGATGGGCTCAGTTTAGGTTTATTGAATTACTGCCTC
CTGCCCACTGCATGTGTATGGGGGGTTCCATTCCCATGTGTGTGAGCCCCCTCCCCTCCCTGGCTGTGCGTAGCTCT
GTCTCTGTCTGTCTCTCTCTCTTTCTCTCCCGCCTCCTCGGTTGGCCGGCCGAAGTTCCTATTCTCTAGAAAGTATAG
GAACTTCTACCGGGTAGGGGAGGCGCTTTTCCCAAGGCAGTCTGGAGCATGCGCTTTAGCAGCCCCGCTGGGCAC
TTGGCGCTACACAAGTGGCCTCTGGCCTCGCACACATTCCACATCCACCGGTAGGCGCCAACCGGCTCCGTTCTTT
GGTGGCCCCTTCGCGCCACCTTCTACTCCTCCCCTAGTCAGGAAGTTCCCCCCCGCCCCGCAGCTCGCGTCGTGCAG
GACGTGACAAATGGAAGTAGCACGTCTCACTAGTCTCGTGCAGATGGACAGCACCGCTGAGCAATGGAAGCGGG
TAGGCCTTTGGGGCAGCGGCCAATAGCAGCTTTGCTCCTTCGCTTTCTGGGCTCAGAGGCTGGGAAGGGGTGGGT
CCGGGGGCGGGCTCAGGGGCGGGCTCAGGGGCGGGGCGGGCGCCCGAAGGTCCTCCGGAGGCCCGGCATTCTG
CACGCTTCAAAAGCGCACGTCTGCCGCGCTGTTCTCCTCTTCCTCATCTCCGGGCCTTTCGACCTGCATCCATCTAGA
TCTCGATCGAGCAGCTGAAGCTTACCGCAGGCTATGAAAAAGCCTGAACTCACCGCGACGTCTGTCGAGAAGTTT
CTGATCGAAAAGTTCGACAGCGTCTCCGACCTGATGCAGCTCTCGGAGGGCGAAGAATCTCGTGCTTTCAGCTTCG
ATGTAGGAGGGCGTGGATATGTCCTGCGGGTAAATAGCTGCGCCGATGGTTTCTACAAAGATCGTTATGTTTATC
GGCACTTTGCATCGGCCGCGCTCCCGATTCCGGAAGTGCTTGACATTGGGGAATTCAGCGAGAGCCTGACCTATT
GCATCTCCCGCCGTGCACAGGGTGTCACGTTGCAAGACCTGCCTGAAACCGAACTGCCCGCTGTTCTGCAGCCGGT
CGCGGAGGCCATGGATGCGATCGCTGCGGCCGATCTTAGCCAGACGAGCGGGTTCGGCCCATTCGGACCGCAAG
GAATCGGTCAATACACTACATGGCGTGATTTCATATGCGCGATTGCTGATCCCCATGTGTATCACTGGCAAACTGT
GATGGACGACACCGTCAGTGCGTCCGTCGCGCAGGCTCTCGATGAGCTGATGCTTTGGGCCGAGGACTGCCCCGA
AGTCCGGCACCTCGTGCACGCGGATTTCGGCTCCAACAATGTCCTGACGGACAATGGCCGCATAACAGCGGTCAT
TGACTGGAGCGAGGCGATGTTCGGGGATTCCCAATACGAGGTCGCCAACATCTTCTTCTGGAGGCCGTGGTTGGC

TTGTATGGAGCAGCAGACGCGCTACTTCGAGCGGAGGCATCCGGAGCTTGCAGGATCGCCGCGGCTCCGGGCGT
ATATGCTCCGCATTGGTCTTGACCAACTCTATCAGAGCTTGGTTGACGGCAATTTCGATGATGCAGCTTGGGCGCA
GGGTCGATGCGACGCAATCGTCCGATCCGGAGCCGGGACTGTCGGGCGTACACAAATCGCCCGCAGAAGCGCGG
CCGTCTGGACCGATGGCTGTGTAGAAGTACTCGCCGATAGTGGAAACCGACGCCCCAGCACTCGTCCGAGGGCAA
AGGAATAGGGGAGGCTAACTGAAGCTTCCCGGGGGTACCAAATTCGTCGACAGATCTAACTTGTTTATTGCAGCT
TATAATGGTTACAAATAAAGCAATAGCATCACAAATTTCACAAATAAAGCATTTTTTTCACTGCATTCTAGTTGTGG
TTTGTCCAAACTCATCAATGTATCTTATGATGTCTGCATATGGAAGTTCCTATTCTCTAGAAAGTATAGGAACTTCG
CGGCCGCGCTGCCAGTTCCAGCTCACGCTTGTAGTGGCTCCGGAAAGCCTCATCCTTGGGCTTCTTGGGATAGAGA
TTTTTGAGCTGAGCAAGATCCCGGATTCGGTCCCCCAGTGAGCGAATGGACAGGTCTTTGGCAGAGAATGGCTGG
ATGTTCTCTATCTGTGGAGAGCCTATGGTAGGAAGGAGACCCTGAGATCCCTCTGTCCGGACTTTCTTCCCCTTCCC
CAACCCCTACCATAAGACCTGTTCTCACTCCACCAAGGCAAGGGAGAGAGGACCTAGGGTGGGGCTCCTGAGGGA
GAGAGACCCACTAGTCTCCGTTCCCACAGAGCTCACAGTGAGAAGGTGAACATTTAGACCACAGAAGGAAGAAG
AGAAGCTGGAAGAACTTCCTGAAGATCAGGATCGGCATCAGGAAGGTCAGGACATTTCATTCCCAGAAGAAACCC
CAGAGGGCACAGGCACAGAGACAGAGGACTGGCTGGGGTGGCCTCACCATCCTGGCCCCGGATGACATGGGCAA
TGGTGATGCCCCCAATCTCTGAGTCGCTGAAGCGGAGGAGAAAGGTTCCGTCGGGCTCATTGAGAAGAAGGCTA
GTAACGTACTGTTTGCTGATGAAGCCAATGATCAGCCTGGCCGGGATGAAGGAGAGGATGGGGCATGGGCAGTG
AGTATGGAGGTGACTGGTGTATGGCTGCTCAGACTACCCAGGGTGGGGACTCACCGGTCAGACCAGTAGCTCCG
GAGACAGCGTTTGGTGAGGTCCAGGACACCATCAAACCACTGCCAAAAGGTGAAGCCACGGCCCAGCAGGATCT
CCTAGGGGGAGAGGGGGAAAGGTGTGAGCCGAGGGAGGGCCAGAGCTGGAGCCTGTTTAGGGCAGGCAGTGA
GCTGTGAGTGCCCGTGGCCAATGCCCTGGCTCACAAATACCACTGAGATCTTTTTCCCTCTGCCAAAAATTATGGG
GACATCATGAAGCCCCTTGAGCATCTGACTTCTGGCTAATAAAGGAAATTTATTTTCATTGCAATAGTGTGTTGGAA
TTTTTTGTGTCTCTCACTCGGAAGGACATATGGGAGGGCAAATCATTTAAAACATCAGAATGAGTATTTGGTTTAG
AGTTTGGCAACATATGCCATATGCTGGCTGCCATGAACAAAGGTGGCTATAAAGAGGTCATCAGTATATGAAACA
GCCCCCTGCTGTCCATTCCTTATTCCATAGAAAAGCCTTGACTTGAGGTTAGATTTTTTTTATATTTTGTTTTGTGTTA
TTTTTTTCTTTAACATCCCTAAAATTTTCCTTACATGTTTTACTAGCCAGATTTTTCCTCCTCTCCTGACTACTCCCAGT
CATAGCTGTCCCTCTTCTCTTATGAAGATCCCTCGACCTGCAGCCCAAGCTTGGCGTAATCATGGTCATAGCTGTTT
CCTGTGTGAAATTGTTATCCGCTCACAATTCCACACAACATACGAGCCGGAAGCATAAAGTGTAAAGCCTGGGGTG
CCTAATGAGTGAGCTAACTCACATTAATTGCGTTGCGCTCACTGCCCGCTTTCCAGTCGGGAAACCTGTCGTGCCA
GCGGATCCGCATCTCAATTAGTCAGCAACCATAGTCCCGCCCCTAACTCCGCCCATCCCGCCCCTAACTCCGCCCAG
TTCCGCCCATTCTCCGCCCCATGGCTGACTAATTTTTTTTATTTATGCAGAGGCCGAGGCCGCCTCGGCCTCTGAGC
TATTCCAGAAGTAGTGAGGAGGCTTTTTTGGAGGCCTAGGCTTTTGCAAAAAGCTAACTTGTTTATTGCAGCTTAT
AATGGTTACAAATAAAGCAATAGCATCACAAATTTCACAAATAAAGCATTTTTTTCACTGCATTCTAGTTGTGGTTT
GTCCAAACTCATCAATGTATCTTATCATGTCTGGATCCGCTGCATTAATGAATCGGCCAACGCGCGGGGAGAGGCG
GTTTGCGTATTGGGCGCTCTTCCGCTTCCTCGCTCACTGACTCGCTGCGCTCGGTCGTTCGGCTGCGGCGAGCGGT
ATCAGCTCACTCAAAGGCGGTAATACGGTTATCCACAGAATCAGGGGATAACGCAGGAAAGAACATGTGAGCAA
AAGGCCAGCAAAAGGCCAGGAACCGTAAAAAGGCCGCGTTGCTGGCGTTTTTCCATAGGCTCCGCCCCCCTGACG
AGCATCACAAAAATCGACGCTCAAGTCAGAGGTGGCGAAACCCGACAGGACTATAAAGATACCAGGCGTTTCCCC
CTGGAAGCTCCCTCGTGCGCTCTCCTGTTCCGACCCTGCCGCTTACCGGATACCTGTCCGCCTTTCTCCCTTCGGGA
AGCGTGGCGCTTTCTCAATGCTCACGCTGTAGGTATCTCAGTTCGGTGTAGGTCGTTCGCTCCAAGCTGGGCTGTG
TGCACGAACCCCCCGTTCAGCCCGACCGCTGCGCCTTATCCGGTAACTATCGTCTTGAGTCCAACCCGGTAAGACA
CGACTTATCGCCACTGGCAGCAGCCACTGGTAACAGGATTAGCAGAGCGAGGTATGTAGGCGGTGCTACAGAGTT
CTTGAAGTGGTGGCCTAACTACGGCTACACTAGAAGGACAGTATTTGGTATCTGCGCTCTGCTGAAGCCAGTTACC
TTCGGAAAAAGAGTTGGTAGCTCTTGATCCGGCAAACAAACCACCGCTGGTAGCGGTGGTTTTTTTGTTTGCAAGC
AGCAGATTACGCGCAGAAAAAAAGGATCTCAAGAAGATCCTTTGATCTTTTCTACGGGGTCTGACGCTCAGTGGA
ACGAAAACTCACGTTAAGGGATTTTGGTCATGAGATTATCAAAAAGGATCTTCACCTAGATCCTTTTAAATTAAAAA
TGAAGTTTTAAATCAATCTAAAGTATATATGAGTAAACTTGGTCTGACAGTTACCAATGCTTAATCAGTGAGGCAC
CTATCTCAGCGATCTGTCTATTTCGTTCATCCATAGTTGCCTGACTCCCCGTCGTGTAGATAACTACGATACGGGAG
GGCTTACCATCTGGCCCCAGTGCTGCAATGATACCGCGAGACCCACGCTCACCGGCTCCAGATTTATCAGCAATAA

ACCAGCCAGCCGGAAGGGCCGAGCGCAGAAGTGGTCCTGCAACTTTATCCGCCTCCATCCAGTCTATTAATTGTTG
CCGGGAAGCTAGAGTAAGTAGTTCGCCAGTTAATAGTTTGCGCAACGTTGTTGCCATTGCTACAGGCATCGTGGT
GTCACGCTCGTCGTTTGGTATGGCTTCATTCAGCTCCGGTTCCCAACGATCAAGGCGAGTTACATGATCCCCCATGT
TGTGCAAAAAAGCGGTTAGCTCCTTCGGTCCTCCGATCGTTGTCAGAAGTAAGTTGGCCGCAGTGTTATCACTCAT
GGTTATGGCAGCACTGCATAATTCTCTTACTGTCATGCCATCCGTAAGATGCTTTTCTGTGACTGGTGAGTACTCAA
CCAAGTCATTCTGAGAATAGTGTATGCGGCGACCGAGTTGCTCTTGCCCGGCGTCAATACGGGATAATACCGCGC
CACATAGCAGAACTTTAAAAGTGCTCATCATTGGAAAACGTTCTTCGGGGCGAAAACTCTCAAGGATCTTACCGCT
GTTGAGATCCAGTTCGATGTAACCCACTCGTGCACCCAACTGATCTTCAGCATCTTTTACTTTCACCAGCGTTTCTGG
GTGAGCAAAAACAGGAAGGCAAAATGCCGCAAAAAAGGGAATAAGGGCGACACGGAAATGTTGAATACTCATAC
TCTTCCTTTTTCAATATTATTGAAGCATTTATCAGGGTTATTGTCTCATGAGCGGATACATATTTGAATGTATTTAGA
AAAATAAACAAATAGGGGTTCCGCGCACATTTCCCCGAAAAGTGCCACCTGG

Blue: left homologous arm
Auburn: knock-in sequence
Red: FRT (flippase recognition target) site
Purple: PGK1-Hygromycin resistance gene
Green: right homologous arm

PCMV-CasRx-U6-sgRNA.NAB2STAT6 sequence:

ATTTTTGTGATGCTCGTCAGGGGGGCGGAGCCTATGGAAAAACGCCAGCAACGCGGCCTTTTTACGGTTCCTGGC
CTTTTGCTGGCCTTTTGCTCACATGTCCTGCAGGCAGCTGCGCGCTCGCTCGCTCACTGAGGCCGCCCGGGCGTCG
GGCGACCTTTGGTCGCCCGGCCTCAGTGAGCGAGCGAGCGCGCAGAGAGGGAGTGGCCAACTCCATCACTAGGG
GTTCCTGCGGCCTCTAGATAGTTATTAATAGTAATCAATTACGGGGTCATTAGTTCATAGCCCATATATGGAGTTCC
GCGTTACATAACTTACGGTAAATGGCCCGCCTGGCTGACCGCCCAACGACCCCCGCCCATTGACGTCAATAATGAC
GTATGTTCCCATAGTAACGCCAATAGGGACTTTCCATTGACGTCAATGGGTGGAGTATTTACGGTAAACTGCCCAC
TTGGCAGTACATCAAGTGTATCATATGCCAAGTACGCCCCCTATTGACGTCAATGACGGTAAATGGCCCGCCTGGC
ATTATGCCCAGTACATGACCTTATGGGACTTTCCTACTTGGCAGTACATCTACGTATTAGTCATCGCTATTACCATG
GTGATGCGGTTTTGGCAGTACATCAATGGGCGTGGATAGCGGTTTGACTCACGGGGATTTCCAAGTCTCCACCCCA
TTGACGTCAATGGGAGTTTGTTTTGGCACCAAAATCAACGGGACTTTCCAAAATGTCGTAACAACTCCGCCCCATT
GACGCAAATGGGCGGTAGGCGTGTACGGTGGGAGGTCTATATAAGCAGAGCTGGTTTAGTGAACCGTCAGATCC
GCTAGCGCTACCGGTCCACCGGTGCCACCATGCCTAAAAAGAAAAGAAAGGTGGGTTCTGGTATCGAGAAGAAG
AAGAGCTTCGCCAAGGGCATGGGAGTGAAGAGCACCCTGGTGTCCGGCTCTAAGGTGTACATGACCACATTTGCT
GAGGGAAGCGACGCCAGGCTGGAGAAGATCGTGGAGGGCGATAGCATCAGATCCGTGAACGAGGGAGAGGCTT
TCAGCGCCGAGATGGCTGACAAGAACGCTGGCTACAAGATCGGAAACGCCAAGTTTTCCCACCCAAAGGGCTACG
CCGTGGTGGCTAACAACCCACTGTACACCGGACCAGTGCAGCAGGACATGCTGGGACTGAAGGAGACACTGGAG
AAGAGGTACTTCGGCGAGTCCGCCGACGGAAACGATAACATCTGCATCCAGGTCATCCACAACATCCTGGATATC
GAGAAGATCCTGGCTGAGTACATCACAAACGCCGCTTACGCCGTGAACAACATCTCCGGCCTGGACAAGGATATC
ATCGGCTTCGGAAAGTTTTCTACCGTGTACACATACGACGAGTTCAAGGATCCAGAGCACCACCGGGCCGCTTTTA
ACAACAACGACAAGCTGATCAACGCCATCAAGGCTCAGTACGACGAGTTCGATAACTTTCTGGATAACCCCAGGCT
GGGCTACTTCGGACAGGCTTTCTTTTCTAAGGAGGGCAGAAACTACATCATCAACTACGGAAACGAGTGTTACGA
CATCCTGGCCCTGCTGAGCGGACTGAGGCACTGGGTGGTGCACAACAACGAGGAGGAGTCTCGGATCAGCCGCA
CCTGGCTGTACAACCTGGACAAGAACCTGGATAACGAGTACATCTCCACACTGAACTACCTGTACGACAGGATCAC
CAACGAGCTGACAAACAGCTTCTCCAAGAACTCTGCCGCTAACGTGAACTACATCGCTGAGACCCTGGGCATCAAC
CCAGCTGAGTTCGCTGAGCAGTACTTCAGATTTTCCATCATGAAGGAGCAGAAGAACCTGGGCTTCAACATCACAA
AGCTGAGAGAAGTGATGCTGGACAGAAAGGATATGTCCGAGATCAGGAAGAACCACAAGGTGTTCGATTCTATC
AGAACCAAGGTGTACACAATGATGGACTTTGTGATCTACAGGTACTACATCGAGGAGGATGCCAAGGTGGCCGCT
GCCAACAAGAGCCTGCCCGACAACGAGAAGTCTCTGAGCGAGAAGGATATCTTCGTGATCAACCTGAGAGGCTCC
TTTAACGACGATCAGAAGGACGCTCTGTACTACGATGAGGCCAACAGGATCTGGAGAAAGCTGGAGAACATCATG
CACAACATCAAGGAGTTCCGGGGAAACAAGACCCGCGAGTACAAGAAGAAGGACGCTCCAAGGCTGCCTAGGAT
CCTGCCTGCTGGAAGGGACGTGAGCGCCTTCAGCAAGCTGATGTACGCCCTGACAATGTTTCTGGACGGAAAGGA
GATCAACGATCTGCTGACCACACTGATCAACAAGTTCGACAACATCCAGTCTTTTCTGAAAGTGATGCCTCTGATCG
GCGTGAACGCTAAGTTCGTGGAGGAGTACGCCTTCTTTAAGGACAGCGCCAAGATCGCTGATGAGCTGCGGCTGA
TCAAGTCCTTTGCCAGGATGGGAGAGCCAATCGCTGACGCTAGGAGAGCTATGTACATCGATGCCATCCGGATCC
TGGGAACCAACCTGTCTTACGACGAGCTGAAGGCTCTGGCCGACACCTTCAGCCTGGATGAGAACGGCAACAAGC
TGAAGAAGGGCAAGCACGGAATGCGCAACTTCATCATCAACAACGTGATCAGCAACAAGCGGTTTCACTACCTGA
TCAGATACGGCGACCCAGCTCACCTGCACGAGATCGCTAAGAACGAGGCCGTGGTGAAGTTCGTGCTGGGACGG
ATCGCCGATATCCAGAAGAAGCAGGGCCAGAACGGAAAGAACCAGATCGACCGCTACTACGAGACCTGCATCGG
CAAGGATAAGGGAAAGTCCGTGTCTGAGAAGGTGGACGCTCTGACCAAGATCATCACAGGCATGAACTACGACC
AGTTCGATAAGAAGAGATCTGTGATCGAGGACACCGGAAGGGAGAACGCCGAGAGAGAGAAGTTTAAGAAGAT
CATCAGCCTGTACCTGACAGTGATCTACCACATCCTGAAGAACATCGTGAACATCAACGCTAGATACGTGATCGGC
TTCCACTGCGTGGAGCGCGATGCCCAGCTGTACAAGGAGAAGGGATACGACATCAACCTGAAGAAGCTGGAGGA
GAAGGGCTTTAGCTCCGTGACCAAGCTGTGCGCTGGAATCGACGAGACAGCCCCCGACAAGAGGAAGGATGTGG
AGAAGGAGATGGCCGAGAGAGCTAAGGAGAGCATCGACTCCCTGGAGTCTGCTAACCCTAAGCTGTACGCCAAC
TACATCAAGTACTCCGATGAGAAGAAGGCCGAGGAGTTCACCAGGCAGATCAACAGAGAGAAGGCCAAGACCGC
TCTGAACGCCTACCTGAGGAACACAAAGTGGAACGTGATCATCCGGGAGGACCTGCTGCGCATCGATAACAAGAC
CTGTACACTGTTCCGGAACAAGGCTGTGCACCTGGAGGTGGCTCGCTACGTGCACGCCTACATCAACGACATCGCC

GAGGTGAACTCCTACTTTCAGCTGTACCACTACATCATGCAGAGGATCATCATGAACGAGAGATACGAGAAGTCTA
GCGGCAAGGTGTCTGAGTACTTCGACGCCGTGAACGATGAGAAGAAGTACAACGATAGACTGCTGAAGCTGCTG
TGCGTGCCTTTCGGATACTGTATCCCACGGTTTAAGAACCTGAGCATCGAGGCCCTGTTCGACCGCAACGAGGCTG
CCAAGTTTGATAAGGAGAAGAAGAAGGTGAGCGGCAACTCCGGTTCTGGTCCCAAGAAGAAGCGCAAGGTGGCG
GCCGCTGATTACAAAGACGATGACGATAAGTAAAGATCCTAAAACTTGTTTATTGCAGCTTATAATGGTTACAAAT
AAAGCAATAGCATCACAAATTTCACAAATAAAGCATTTTTTTCACTGCATTCTAGTTGTGGTTTGTCCAAACTCATCA
ATGTATCTTATCATGTCTGGATCGCGGCCGAGGGCCTATTTCCCATGATTCCTTCATATTTGCATATACGATACAAG
GCTGTTAGAGAGATAATTGGAATTAATTTGACTGTAAACACAAAGATATTAGTACAAAATACGTGACGTAGAAAG
TAATAATTTCTTGGGTAGTTTGCAGTTTTAAAATTATGTTTTAAAATGGACTATCATATGCTTACCGTAACTTGAAAG
TATTTCGATTTCTTGGCTTTATATATCTTGTGGAAAGGACGAAACACCGCAAGTAAACCCCTACCAACTGGTCGGG
GTTTGAAACTACCCATCTGTTCAGCTGCGAGAGGTGGCTCAAGTAAACCCCTACCAACTGGTCGGGGTTTGAAACT
TTTTTTGAATTCTGATGCGGTGGCGGCCGCAGGAACCCCTAGTGATGGAGTTGGCCACTCCCTCTCTGCGCGCTCG
CTCGCTCACTGAGGCCGGGCGACCAAAGGTCGCCCGACGCCCGGGCTTTGCCCGGGCGGCCTCAGTGAGCGAGC
GAGCGCGCAGCTGCCTGCAGGGGCGCCTGATGCGGTATTTTCTCCTTACGCATCTGTGCGGTATTTCACACCGCAT
ACGTCAAAGCAACCATAGTACGCGCCCTGTAGCGGCGCATTAAGCGCGGCGGGTGTGGTGGTTACGCGCAGCGT
GACCGCTACACTTGCCAGCGCCTTAGCGCCCGCTCCTTTCGCTTTCTTCCCTTCCTTTCTCGCCACGTTCGCCGGCTT
TCCCCGTCAAGCTCTAAATCGGGGGCTCCCTTTAGGGTTCCGATTTAGTGCTTTACGGCACCTCGACCCCAAAAAAC
TTGATTTGGGTGATGGTTCACGTAGTGGGCCATCGCCCTGATAGACGGTTTTTCGCCCTTTGACGTTGGAGTCCAC
GTTCTTTAATAGTGGACTCTTGTTCCAAACTGGAACAACACTCAACTCTATCTCGGGCTATTCTTTTGATTTATAAGG
GATTTTGCCGATTTCGGTCTATTGGTTAAAAAATGAGCTGATTTAACAAAAATTTAACGCGAATTTTAACAAAATAT
TAACGTTTACAATTTTATGGTGCACTCTCAGTACAATCTGCTCTGATGCCGCATAGTTAAGCCAGCCCCGACACCCG
CCAACACCCGCTGACGCGCCCTGACGGGCTTGTCTGCTCCCGGCATCCGCTTACAGACAAGCTGTGACCGTCTCCG
GGAGCTGCATGTGTCAGAGGTTTTCACCGTCATCACCGAAACGCGCGAGACGAAAGGGCCTCGTGATACGCCTAT
TTTTATAGGTTAATGTCATGATAATAATGGTTTCTTAGACGTCAGGTGGCACTTTTCGGGGAAATGTGCGCGGAAC
CCCTATTTGTTTATTTTTCTAAATACATTCAAATATGTATCCGCTCATGAGACAATAACCCTGATAAATGCTTCAATA
ATATTGAAAAAGGAAGAGTATGAGTATTCAACATTTCCGTGTCGCCCTTATTCCCTTTTTTGCGGCATTTTGCCTTCC
TGTTTTTGCTCACCCAGAAACGCTGGTGAAAGTAAAAGATGCTGAAGATCAGTTGGGTGCACGAGTGGGTTACAT
CGAACTGGATCTCAACAGCGGTAAGATCCTTGAGAGTTTTCGCCCCGAAGAACGTTTTCCAATGATGAGCACTTTT
AAAGTTCTGCTATGTGGCGCGGTATTATCCCGTATTGACGCCGGGCAAGAGCAACTCGGTCGCCGCATACACTATT
CTCAGAATGACTTGGTTGAGTACTCACCAGTCACAGAAAAGCATCTTACGGATGGCATGACAGTAAGAGAATTAT
GCAGTGCTGCCATAACCATGAGTGATAACACTGCGGCCAACTTACTTCTGACAACGATCGGAGGACCGAAGGAGC
TAACCGCTTTTTTGCACAACATGGGGGATCATGTAACTCGCCTTGATCGTTGGGAACCGGAGCTGAATGAAGCCAT
ACCAAACGACGAGCGTGACACCACGATGCCTGTAGCAATGGCAACAACGTTGCGCAAACTATTAACTGGCGAACT
ACTTACTCTAGCTTCCCGGCAACAATTAATAGACTGGATGGAGGCGGATAAAGTTGCAGGACCACTTCTGCGCTCG
GCCCTTCCGGCTGGCTGGTTTATTGCTGATAAATCTGGAGCCGGTGAGCGTGGAAGCCGCGGTATCATTGCAGCA
CTGGGGCCAGATGGTAAGCCCTCCCGTATCGTAGTTATCTACACGACGGGGAGTCAGGCAACTATGGATGAACGA
AATAGACAGATCGCTGAGATAGGTGCCTCACTGATTAAGCATTGGTAACTGTCAGACCAAGTTTACTCATATATAC
TTTAGATTGATTTAAAACTTCATTTTTAATTTAAAAGGATCTAGGTGAAGATCCTTTTTGATAATCTCATGACCAAAA
TCCCTTAACGTGAGTTTTCGTTCCACTGAGCGTCAGACCCCGTAGAAAAGATCAAAGGATCTTCTTGAGATCCTTTT
TTTCTGCGCGTAATCTGCTGCTTGCAAACAAAAAAACCACCGCTACCAGCGGTGGTTTGTTTGCCGGATCAAGAGC
TACCAACTCTTTTTCCGAAGGTAACTGGCTTCAGCAGAGCGCAGATACCAAATACTGTTCTTCTAGTGTAGCCGTAG
TTAGGCCACCACTTCAAGAACTCTGTAGCACCGCCTACATACCTCGCTCTGCTAATCCTGTTACCAGTGGCTGCTGC
CAGTGGCGATAAGTCGTGTCTTACCGGGTTGGACTCAAGACGATAGTTACCGGATAAGGCGCAGCGGTCGGGCT
GAACGGGGGGTTCGTGCACACAGCCCAGCTTGGAGCGAACGACCTACACCGAACTGAGATACCTACAGCGTGAG
CTATGAGAAAGCGCCACGCTTCCCGAAGGGAGAAAGGCGGACAGGTATCCGGTAAGCGGCAGGGTCGGAACAG
GAGAGCGCACGAGGGAGCTTCCAGGGGGAAACGCCTGGTATCTTTATAGTCCTGTCGGGTTTCGCCACCTCTGAC
TTGAGCGTCG
Red: AAV2 ITR

Green: CMV promoter
Purple: SV40 NLS
Auburn: CasRx
Orange: U6 promoter
Yellow: sgRNA.NAB2STAT6

Supplementary Figure S1

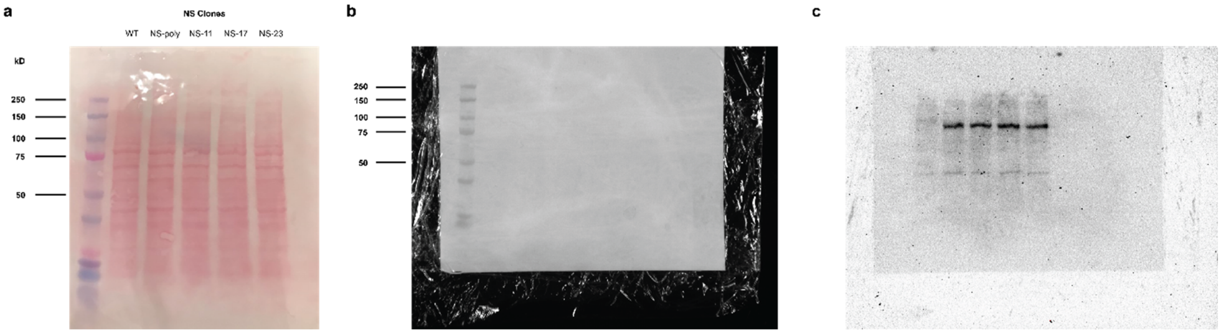

Supplementary Figure S1. Original data for Western blot. (a) Ponceau staining of the whole cell lysates prepared from HCT116 wild type and NAB2-STAT6 fusion cell lines. **(b) F**ull Western blot image (bright field). **(c)** Full Western blot image (chemiluminescence).

Supplementary Figure S2

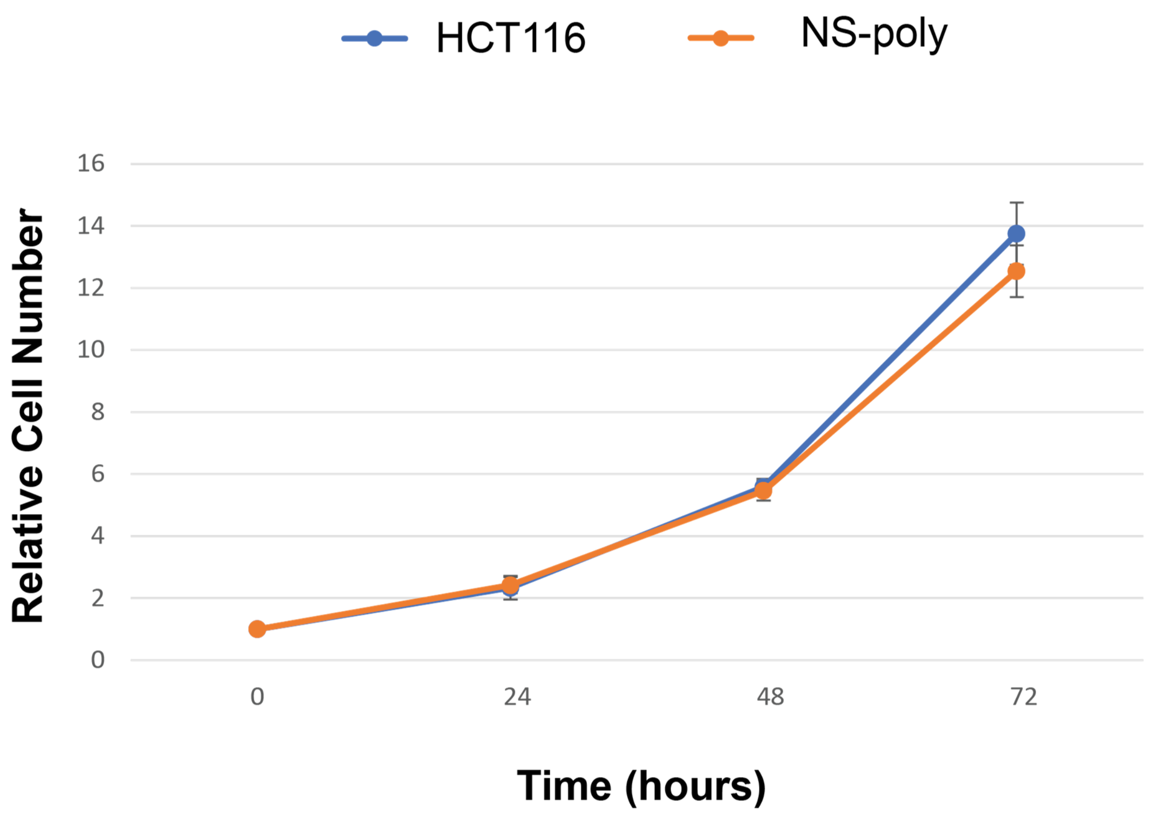

Supplementary Figure S2. Cell proliferation assays for wild type HCT116 and NS-poly cells. No significantly different proliferation rate was observed between wild type HCT116 and NS-poly cells.

Supplementary Figure S3

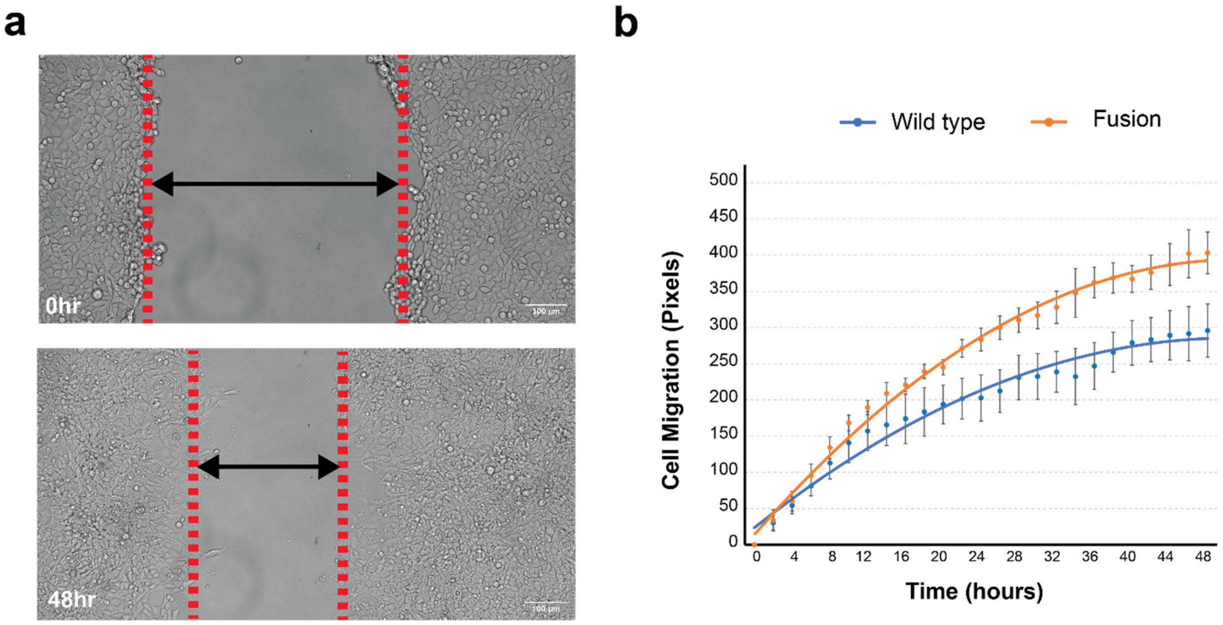

Supplementary Figure S3. ***In vitro*** **characterization of NS-poly cell model. (a)** Representative wound healing assay images for wild type HCT116 cells at 0 and 48 hours. **(b)** Wound healing assay results for both wild type HCT116 and NS-poly cells. The NS-poly cells showed higher motility rate compared to wild type HCT116.

Supplementary Table S1: Summary sequencing statistics of the RNA-seq assay.

Supplementary Table S2: Primers used in this study.

Supplementary Table S3: Wound healing assays using HCT116 and NS-poly cells.

Supplementary Table S4: Differentially expressed genes between wild type HCT116 and NAB2-STAT6 fusion cells from RNA-seq. Negative values in the log2FoldChange reflected fold changes observed in the control samples (HCT116 parental cells) compared to experimental samples (our engineered NAB2-STAT6 fusion cells). Therefore, negative values indicated that expression levels of such genes were elevated in the engineered NAB2-STAT6 fusion cells.

Supplementary Table S5: Differentially expressed genes used for PANTHER signaling pathway analysis.

Supplementary Table S6: NAB2-STAT6 fusion-related signaling pathways identified by the PANTHER classification system.

Supplementary Table S7: NAB2-STAT6 fusion-specific ASOs with 2'-O-methoxyethyl modifications.

prepDE.py: The python script used to quantify both expressed genes and transcripts.

DESeq2.r: The R script used to identify differentially expressed genes and prepare for the corresponding heatmap.
